# Beyond the hype: Navigating bias in AI-driven cancer detection

**DOI:** 10.18632/oncotarget.28665

**Published:** 2024-11-07

**Authors:** Yashbir Singh, Heenaben Patel, Diana V. Vera-Garcia, Quincy A. Hathaway, Deepa Sarkar, Emilio Quaia

**Keywords:** AI bias, cancer detection, healthcare disparities, medical ethics, AI regulation

In recent years, the integration of artificial intelligence (AI) into healthcare has been heralded as a revolutionary force, particularly in cancer detection. Headlines tout AI systems outperform human radiologists in identifying tumors, promising a future where cancer diagnoses are faster, more accurate, and universally accessible. However, as we stand on the cusp of this AI-driven medical revolution, it is crucial to look beyond the hype and address a significant challenge: bias in AI-driven cancer detection systems. There is a growing use of AI technology to identify cancer in early stages, from mammograms, CT scan images, or biopsy images. The applications of deep learning algorithms are expanding and new approaches have demonstrated remarkable capabilities in cancer screening, diagnosis, risk prediction, prognosis, treatment strategy, response assessment, and follow-up [1]. These advancements have sparked hope for earlier cancer detection, improved treatment decisions and planning, and reduced morbidity and mortality.

As we eagerly adopt Al models, we need to take a moment to think about the potential biases that they may contain. It is important to remark that these models are not the solution for every cancer issue, and they may have inherent limitations. The accuracy of an AI-based model relies on the data on which the model has been trained. This means that if the initial datasets are not representative of the population where it will be used, it would ultimately impact the performance of the test and affect generalizability. For example, if an AI model is trained on Caucasian patients, it may struggle to detect skin cancer in patients with darker skin accurately, leading to missed diagnoses or false positives [2]. Further, the impact of a population’s culture (e.g., genetics, diets, traditions, access to healthcare) can result in various presentations and incidence rates of a specific disease, which may be difficult to predict if an AI model does not have adequate training data. Most algorithms learn from historical datasets without existing disparities in healthcare, and if these datasets are not diverse and representative of all populations, the resulting AI systems may perform poorly for underrepresented groups [3]. As discussed, the bias in AI is not limited to racial disparities. Multiple factors such as socioeconomic status, gender, age, internet access, and geographic location can influence the quality and availability of medical data and the performance of AI systems. An AI system trained on data from well-funded urban hospitals may perform weakly when applied in rural or low-resource settings [4].

The consequences of biased AI in cancer detection could cost billions of dollars to the healthcare system and worsen healthcare expenditures. Misdiagnoses can lead to unnecessary treatments or delayed interventions. Missed opportunities for early detection for cancers that require prompt treatment such as small cell lung cancer, pancreatic cancer, or aggressive melanoma can lead to severe consequences that patients and families would face. Furthermore, if healthcare providers rely too heavily on these AI tools without understanding or knowing their limitations, it could erode trust in medical AI between medical providers and patients [5]; this could hinder the adoption of Al rather than allow it to be a powerful and beneficial tool. Addressing bias in AI-driven cancer detection is not just a technical challenge; it’s an ethical imperative. As we develop and deploy these systems, we must prioritize fairness and transparency within a multi-faceted approach:

1. Diverse and Representative Data: We must make concerted efforts to collect diverse datasets representing all populations, such as age, gender, race, socioeconomic status, ethnicity, and genetic differences. This may involve targeted data collection initiatives in underserved communities and collaboration across global healthcare systems.
2. Rigorous Testing and Validation: AI systems should be rigorously tested across different populations and healthcare settings before deployment. This includes evaluating performance disparities across various demographic groups and geographical locations.
3. Transparency and Explainability: Developers should strive to create AI systems that can explain their decision-making processes in depth. This transparency is crucial for building trust and allowing healthcare providers to understand and mitigate potential biases. To be effective in clinical practice, algorithms should provide patients and clinicians with useful information that can help shape modifiable behaviors and improve health outcomes.
4. Interdisciplinary Collaboration: The development of AI in healthcare should involve not just data scientists and clinicians but also ethicists, sociologists, and patient advocates. This diverse input can help identify and address potential biases from multiple perspectives.
5. Ongoing Monitoring and Refinement: Once deployed, AI systems should be continuously monitored for performance across different populations. Regular audits and updates can help identify and correct biases that emerge over time.
6. Education and Training: Healthcare providers need to be educated about the capabilities and limitations of AI systems, including potential biases. This understanding is crucial for the appropriate use and interpretation of AI-generated insights.

As we navigate the exciting frontier of AI in cancer detection, we must remain vigilant that its integration into healthcare must be guided by ethical principles. The promise of AI in cancer detection is too great to be undermined by unchecked biases. By acknowledging and actively addressing these challenges, we can work towards a future where AI truly enhances cancer care for all patients, regardless of race, gender, age, or socioeconomic status.

## Expanding the scope: From detection to treatment

While much of the focus on AI in oncology has been on detection, it’s crucial to recognize that the impact of biased AI systems extend far beyond the initial diagnosis. As AI increasingly identifies targeted therapies based on genetics and molecular markers, makes survival predictions, and aids in treatment decisions, the potential for bias to influence patient outcomes can effect multiple levels. Consider, for instance, the use of AI in predicting patient responses to different treatment regimens. Suppose the AI model has been trained on missing data, a reduced sample size, or misclassified data for a specific population. In that case, it may provide misleading and inaccurate recommendations leading to suboptimal treatment choices and compromised patient care [6]. Furthermore, as AI systems start to be involved in clinical trials, biases in these algorithms could continue the trend of minority groups being underrepresented in medical research. This not only restricts the applicability of research findings but also denies underrepresented populations the potential benefits of advanced treatments.

## The role of regulatory bodies and policy makers

Addressing bias in AI-driven cancer care is not solely the responsibility of researchers and healthcare providers. Regulatory bodies and policymakers have a crucial role to play in ensuring the ethical development and deployment of this technology. The U.S. Food and Drug Administration (FDA) has already begun to grapple with those challenges by releasing a regulation framework for AI-based medical devices [7]. However, as the field rapidly evolves, there is a need for more comprehensive and nuanced regulatory frameworks that specifically address issues of bias and fairness. Policymakers should consider mandating diversity in clinical trials for AI-based medical technologies, similar to existing requirements for drug trials. They should also explore ways to incentivize the development of AI systems that demonstrate fairness across diverse populations, perhaps through expedited review processes or other regulatory incentives.

## A call to action: Collaborative efforts for equitable AI

Addressing bias in AI-driven cancer detection and treatment is not a challenge that any single entity can solve. It requires a collaborative effort involving stakeholders from across the healthcare ecosystem:

1. Researchers and Developers: Must prioritize fairness and inclusivity in AI design, actively seeking out diverse datasets and implementing robust bias detection and mitigation strategies.
2. Healthcare Providers: There is an increased need to develop AI literacy and understand both the potential and limitations of these tools. They should actively participate in the development and refinement of AI systems, providing crucial clinical context and feedback.
3. Patients and Advocacy Groups: Should be involved in the AI development process, ensuring that diverse patient perspectives are considered and that AI systems address real-world needs and concerns.
4. Regulatory Bodies: Must evolve frameworks to effectively oversee AI in healthcare, balancing innovation with patient safety and fairness.
5. Policymakers: Should create incentives for the development of equitable AI systems and support initiatives to increase diversity in both AI development and clinical trials.
6. Ethicists and Social Scientists: Play a crucial role in identifying potential ethical pitfalls and societal implications of AI in healthcare, helping to guide responsible development and deployment.

As we continue to push the boundaries of what’s possible with AI in cancer care, we must ensure that our ethical considerations match our technological progress. The goal should not merely be to create AI systems that are more accurate than humans but to develop technologies that are fundamentally fair and beneficial to all patients.

By confronting the challenge of bias, we can work towards a future where AI truly democratizes access to high-quality cancer care, reducing disparities rather than reinforcing them. This is the promise of AI in oncology that we must strive to fulfill – a future where cutting-edge technology benefits every patient, regardless of their background.
